# Does the Anti-Mullerian Hormone Decline Rate Improve the Prediction of Age at Menopause?

**DOI:** 10.3389/fendo.2021.727229

**Published:** 2021-09-16

**Authors:** Fahimeh Ramezani Tehrani, Ali Sheidaei, Faezeh Firouzi, Maryam Tohidi, Fereidoun Azizi, Samira Behboudi-Gandevani

**Affiliations:** ^1^Reproductive Endocrinology Research Center, Research Institute for Endocrine Sciences, Shahid Beheshti University of Medical Sciences, Tehran, Iran; ^2^Pathology Department, Shahid Beheshti University of Medical Sciences, Tehran, Iran; ^3^Prevention of Metabolic Disorders Research Center, Research Institute for Endocrine Sciences, Shahid Beheshti University of Medical Sciences, Tehran, Iran; ^4^Endocrine Research Center, Research Institute for Endocrine Sciences, Shahid Beheshti University of Medical Sciences, Tehran, Iran; ^5^Faculty of Nursing and Health Sciences, Nord University, Bodø, Norway

**Keywords:** anti-Mullerian hormone, menopause, proportional hazard Cox regression, Tehran Lipid and Glucose Study, time-dependent Cox regression

## Abstract

**Objectives:**

There are controversial studies investigating whether multiple anti-Mullerian hormone (AMH) measurements can improve the individualized prediction of age at menopause in the general population. This study aimed to reexplore the additive role of the AMH decline rate in single AMH measurement for improving the prediction of age at physiological menopause, based on two common statistical models for analysis of time-to-event data, including time-dependent Cox regression and Cox proportional-hazards regression models.

**Methods:**

A total of 901 eligible women, aged 18–50 years, were recruited from the Tehran Lipid and Glucose Study (TLGS) population and followed up every 3 years for 18 years. The serum AMH level was measured at the time of recruitment and twice after recruitment within 6-year intervals using the Gen II AMH assay. The added value of repeated AMH measurements for the prediction of age at menopause was explored using two different statistical approaches. In the first approach, a time-dependent Cox model was plotted, with all three AMH measurements as time-varying predictors and the baseline age and logarithm of annual AMH decline as time-invariant predictors. In the second approach, a Cox proportional-hazards model was fitted to the baseline data, and improvement of the complex model, which included repeated AMH measurements and the logarithm of the AMH annual decline rate, was assessed using the C-statistic.

**Results:**

The time-dependent Cox model showed that each unit increase in the AMH level could reduce the risk of menopause by 87%. The Cox proportional-hazards model also improved the prediction of age at menopause by 3%, according to the C-statistic. The subgroup analysis for the prediction of early menopause revealed that the risk of early menopause increased by 10.8 with each unit increase in the AMH annual decline rate.

**Conclusion:**

This study confirmed that multiple AMH measurements could improve the individual predictions of the risk of at physiological menopause compared to single AMH measurements. Different alternative statistical approaches can also offer the same interpretations if the essential assumptions are met.

## Introduction

Menopause is a unique event in a woman’s life, with significant physical, psychological, and social effects on her health (1). In this phenomenon, the main biological change is a reduction of the primordial follicle pool, leading to menopause at the mean age of 51 years (2). Overall, the accurate estimation of age at menopause can enable women to make informed decisions about their future fertility and desired family size. Also, strategies can be devised for reducing the short- or long-term health risks of early and late menopause.

So far, many candidate markers have been identified to estimate the age at menopause. Considering the strong association between women’s age, number of primordial and growing follicles, and serum anti-Mullerian hormone (AMH) levels, efforts have been made for the individualized prediction of age at menopause, based on the serum concentrations of AMH. Generally, this hormone is a member of the transforming growth factor-β (TGF-β) family, which is produced by follicular granulosa cells of small growing follicles.

So far, several studies have been performed to examine the association between age at menopause and the serum concentration of AMH using various mathematical models (3–8). However, the serum AMH level appears to have limited precision at an individual level, particularly for women with an early menopause. Besides, there is uncertainty regarding the added value of repeated AMH measurements for improving the prediction of age at menopause. It is well documented that the ovarian reserve status is not constant throughout one’s life and is influenced by different intrinsic and extrinsic factors (9–14); therefore, it is hypothesized that serial AMH measurements may indirectly estimate these changes.

Controversial results have been reported in studies investigating whether multiple AMH measurements can improve the individualized prediction of age at menopause in the general population (15–19). Apart from different complex and dynamic factors, such as ethnicity, age range, duration of follow-up, and study design that can cause discrepancy between studies, the statistical models used for the analysis of data may be also influential. Although Cox models have been widely used for data analysis, the absence of essential assumptions and technical intricacies may lead to bias in estimations of regression coefficients in these models.

In our previous study (19), we reported that prediction of age at menopause could be improved by multiple AMH measurements, using an accelerated failure time model with the Weibull distribution, while another well-designed study reported no added value for repeated AMH measurements based on the Cox proportional-hazards models with time-varying covariates (16). Therefore, in the present study, we aimed to reanalyze our dataset using the most common Cox regression models, based on the essential assumptions to reexplore the added value of AMH decline rate to single AMH measurements for improving the prediction of age at physiological menopause.

## Materials and Methods

This study was approved by the ethics committee of the Research Institute for Endocrine Sciences, and a written informed consent was obtained from all subjects before initiation of the study (No. IR.SBMU.ENDOCRINE.REC.1395.347).

### Study Participants

The participants of this study were selected from the population of Tehran Lipid and Glucose Study (TLGS), which is an ongoing, long-term, population-based prospective study, initiated in 1998 to evaluate the risk factors for non-communicable disorders in an urban population. A more exhaustive description of the TLGS is published elsewhere (20).

The present study was performed on all female TLGS participants, aged 18–50 years, with regular and predictable menstrual cycles and proven natural fertility at the beginning of the study. The exclusion criteria were as follows: being currently pregnant; a history of hysterectomy, oophorectomy, or any type of ovarian surgery; premature menopause before the age of 40 years; all those women age < 40 at the end of study; current use of psychotropic or hormonal medications, including hormonal contraceptives or hormone therapies; and serious diseases known to interfere with the ovarian function, such as breast or endometrial cancer and endocrine disorders.

### Study Design

The cohort was followed up for 18 years after enrollment in the study (1998–2001). They completed a standard questionnaire (including the reproductive history and date of the last menstrual cycle) in face-to-face interviews with trained staff once at baseline and every 3 years after enrollment in the study in the follow-up visits. Besides, a general practitioner performed the general anthropometric measurements and physical examinations. After 12–14 h overnight fasting, blood samples were obtained from the subjects between 07:00 a.m. and 09:00 a.m., the plasma was separated in a refrigerated centrifuge at 3,000 rpm for 10 min, and the sera were stored at -80°C for further assessments.

### Study Variables

According to the World Health Organization (WHO) classification, natural menopause occurs after 12 consecutive months of amenorrhea, for which there is no other obvious pathological or physiological cause (21). The date of menopause was defined as a point in time 12 months after a woman’s last period.

The AMH level was measured at three time points (baseline, third and sixth follow-up), using a two-site enzyme immunoassay with Gen II Kit (Beckman Coulter, Inc., Fullerton, CA, USA) and a Sunrise ELISA reader (Tecan Co., Salzburg, Austria). A modified protocol was used for measuring the AMH levels, as the original Gen Kit may underestimate the serum AMH level (22). A single experienced laboratory technician performed all AMH measurements simultaneously at the same laboratory. The AMH Gen II control A79766 was used at two concentrations to monitor the accuracy of assays. The intra- and inter-assay coefficients of variation were estimated at 1.9% and 2.0%, respectively.

### Statistical Method

For elimination of outlier effects, we used 95% trim data. Initially, we defined three age categories: 18–30, 31–40, and 41–50 years. For all these categories, the 2.5th and 97.5th percentiles of AMH values were calculated, and any observation beyond these values was removed. Moreover, for a survival analysis, we considered the observation time to be the most recent information about the age of women who did not experience menopause or the menopausal age of women who reached menopause during the study. Besides, the storage time for AMH was determined as the time interval between sample collection and AMH measurements. The AMH annual decline rate was also calculated based on the difference between the first and the last measured AMH, divided by the interval between these two measurements.

To assess the added value of a second AMH measurement, we employed a stepwise approach. First, the baseline AMH level was considered as the predictor of age at menopause in a Cox proportional-hazards model. In the next model, the second AMH measurement was integrated. In Model 3, the logarithm of the annual AMH decline rate was included instead of the second AMH measurement. Finally, Model 4 included both the second AMH measurement and logarithm of annual AMH decline. All these models were adjusted by age at baseline and AMH storage time of the samples. Nevertheless, a third AMH measurement was not considered in the analysis due to lack of follow-up after this point.

The predictive power of the models was evaluated by the C-statistic. Moreover, the proportional hazards (PH) assumption for the Cox regression models was examined, using the cox.zph function in the survival package of R software (15). This function examines the proportionality of all predictors included in the model by creating interactions with time. A small p-value can indicate a violation of the PH assumption. Besides, we generated a plot for each AMH measurement and logarithm of annual AMH decline rate in the models. If the PH assumption is met, a slope of zero is inevitable in the plot. On the other hand, a slope that significantly diverges from zero suggests a violation of the PH assumption (15).

Generally, correlations between predictors, including different AMH measurements and the AMH annual decline rate, may cause multicollinearity issues. Therefore, we used the variance inflation factor (VIF) index to quantify the level of multicollinearity. A VIF of 1 suggests that there is no correlation between the predictor of interest and the remaining predictive variables; therefore, variance in this predictor is not inflated at all. As a general rule, VIFs exceeding 4 require further investigation, while VIFs exceeding 10 suggest serious multicollinearity requiring a model correction (23).

As an alternative approach, we used the time-dependent Cox model to evaluate the predictive power of all AMH measurements for age at menopause. Overall, when the PH assumption is violated, it is suggested to use extended Cox models, such as time-dependent Cox models. We considered two scenarios for modeling the relationship between the AMH level and age at menopause. In the first approach, we used the time-dependent Cox model to evaluate the predictive power of all three AMH measurements for age at menopause. The logarithm transformation of these values was included in the model, and the observation time and AMH annual decline rate were defined as described earlier. The AMH levels and AMH storage time were also entered in the models as time-varying predictors, while the baseline age and logarithm of annual AMH decline rate were considered as time-invariant variables.

To explore the value of repeated AMH measurements for the prediction of early menopause (menopause <45 years), a subgroup analysis was performed, including an observation time <45 years. Statistical analyses were performed in R Version 4.0.2 (R Foundation for Statistical Computing, Vienna, Austria) (24).

## Results

A total of 901 TLGS participants met the eligibility criteria and were observed for a median follow-up of 13 years (interquartile range [IQR]: 9–16); overall, 522 women reached menopause during the study. The mean (SD) age of the subjects was 36 (6.0), 43 (5.9), and 50 (5.2) years at baseline and in the third and sixth follow-ups, respectively, and the mean body mass index (BMI) was 27 (4.7), 28 (4.4), and 29 (4.8) kg/m^2^, respectively (**Table 1**).

**Table 1:** Characteristics of study participants.

	Baseline	Third follow-up	Sixth follow-up
	N = 901	N = 859	N = 638
Age (year), mean (SD)	36 (6.0)	43 (5.9)	50 (5.2)
<30 (year), n (%)	172 (19.09)	131 (15.25)	-
31–40 (year), n (%)	447 (49.61)	290 (33.76)	-
>40 (year), n (%)	282 (31.30)	438 (50.99)	638 (100)
BMI (kg/m^2^)	27 (4.7)	28 (4.4)	29 (4.8)
<25 (kg/m^2^), n (%)	290 (32.19)	280 (32.60)	123 (19.28)
25–29 (kg/m^2^), n (%)	373 (41.40)	355 (41.33)	233 (36.52)
≥30 (kg/m^2^), n (%)	230 (25.53)	216 (25.15)	171 (26.80)
WC (cm), mean (SD)	85 (11)	88 (12)	85 (11)
WHR, mean (SD)	0.82 (0.07)	0.82 (0.07)	0.82 (0.06)
Parity, n (%)	2.5 (1.5)	2.5 (1.5)	2.0 (1.5)
Central obesity ^*^, n (%)	377 (42.6)	356 (42.23)	386 (61.86)
AMH ^**^, (ng/ml)	1.5 (1.5)	0.66 (0.75)	0.32 (0.44)
Storage time for AMH, ^**^ (year)	8.7 (0.5)	4.2 (0.9)	1 (0.8)
Menopause, N (%)	0 (0)	194 (22)	522 (82)

Values are given as mean (standard deviation), or number (percentage), as indicated.

BMI, body mass index; WC, waist circumference; WHR, waist to hip ratio; AMH, anti-Mullerian hormone.

^*^Central obesity is defined as a waist circumference ≥ 88 cm.

^**^Only reported for those not reached menopause.

**Figure 1:**
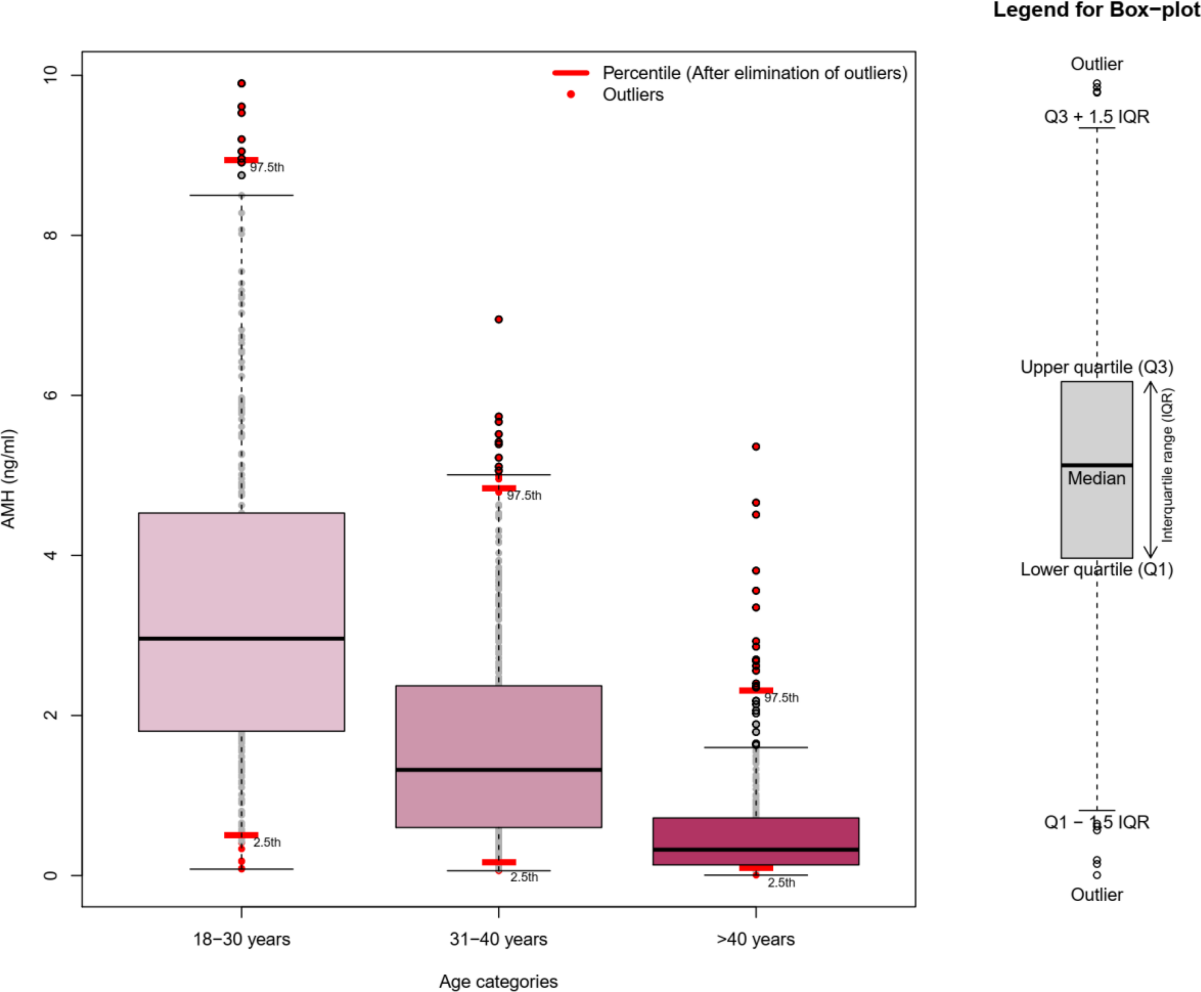
Comparing trim data outlier detection with the box plot approach across age categories.

Moreover, the mean (SD) serum AMH levels were 1.5 (1.5), 0.66 (0.75), and 0.32 (0.44) ng/ml at baseline and in the third and sixth follow-ups, respectively. Also, the mean age-specific AMH annual decline rates for the 5th, 25th, 50th, 75th, and 95th percentiles of our population were estimated at 0.006, 0.032, 0.079, 0.14, and 0.29 ng/ml, respectively. **Figure 1** compares our method for identification of outliers (trimming the dataset) with graphic box plots as the most common method. It was found that eliminated data in our approach (presented as red dots) were much less than the common approach.

**Table 2** compares the C-statistics for the Cox proportional-hazards model, including single baseline AMH measurements, and advanced models, including repeated AMH measurements and logarithm of annual AMH decline rate (**Table 2**). In Model 1 with the baseline AMH values, the risk of experiencing menopause during the follow-ups decreased by 57% with each unit (ng/mL) increase in the baseline serum AMH levels in women of the same age (C-statistic = 0.71). AMH measurements at second time points decreased the C-statistic to 0.70 in Model 2, and adding the AMH annual decline rate to Model 1 improved the C-statistic by 3% (0.74). In other words, with each unit increase in logarithm of annual AMH decline rate (ng/mL/y), the risk of menopause increased by 37%.

**Table 2:** Summary of statistics for the proportional hazard Cox regression approach (include test of proportional hazards assumption) for prediction of menopause/early menopause according to single/repeated AMH measurements.

Variable	Menopause prediction during follow-up				Prediction of early menopause^€^			
	HR (95% CI)	p value PH test	VIF	C-statistic (95% CI)	HR (95% CI)	p value PH test	VIF	C-statistic (95% CI)
Model 1*				0.71 (0.69–0.73)				0.71 (0.69–0.73)
Global PH assumption		0.002				0.011		
Baseline AMH value	**0.43 (0.37–0.50)**	0.013	1.40		**0.44 (0.38–0.52)**	0.003	1.27	
Model 2^**^				0.70 (0.68–0.72)				0.73 (0.69–0.78)
Global PH assumption		0.006				0.75		
Baseline AMH	**0.49 (0.39–0.61)**	0.173	3.03		**0.49 (0.33–0.72)**	0.70	2.75	
Second AMH value	0.78 (0.49–1.25)	0.011	2.88		0.71 (0.35–1.45)	0.23	2.83	
Model 3^**^				0.74 (0.71–0.76)				0.75 (0.71–0.79)
Global PH assumption		<0.001				0.028		
Baseline AMH value	**0.29 (0.23–0.37)**	0.177	2.60		**0.22 (0.14–0.34)**	0.440	2.58	
Log (annual AMH decline rate^£^)	**1.37 (1.20–1.56)**	0.056	1.92		**2.33 (1.49–3.63)**	0.101	2.61	
Model 4^**^				0.73 (0.70–0.75)				0.75 (0.71–0.80)
Global PH assumption		<0.001				0.197		
Baseline AMH value	**0.19 (0.12–0.30)**	0.122	9.90		**0.06 (0.02–0.16)**	0.341	12.91	
Second AMH value	**2.34 (1.22–4.49)**	0.004	5.55		**5.95 (1.74–20.36)**	0.094	7.63	
Log (annual AMH decline rate)	**1.56 (1.31–1.86)**	0.044	3.11		**4.34 (2.35–8.02)**	0.255	4.17	

^*^Model 1: adjusted by age at baseline and storage time of baseline AMH.

^**^Models 2, 3, and 4: adjusted by age at baseline, storage times of baseline, and second AMH measures.

Significant results are reported in bold.

AMH, anti-Mullerian hormone; HR, hazard ratio; VIF, variation inflation factor; PH, proportional hazard.

^£^Annual AMH decline rate was calculated according (last AMH-baseline AMH)/time intervals (years), reported as AMH (ng/ml/year).

^€^Early menopause was defined as menopause occurred at age 40–45 years.

For further clarification, assume that we want to evaluate the time to menopause in two females with similar age. The mean baseline level of AMH was 1.8 ng/ml in these two cases. The second measurement showed that the AMH level of subject 1 reduced to 1.7 ng/ml after 1 year, while the AMH level reduced to 1.53 ng/ml in subject 2. Overall, the risk of earlier menopause in subject 2 was 37% higher than in subject 1; interestingly, the risk of early menopause was 2.33 times higher in subject 2. However, this key finding would have been missed if we had not conducted the second measurements.

Both the second AMH measurements and the logarithm of annual AMH decline rate decreased the C-statistic to 0.73 in Model 4. However, according to the C-statistic, the best model for predicting menopause/early menopause was Model 3. Based on the PH assumption, none of the models could satisfy all of the included variables considering the p-values < 0.05, except for the baseline AMH measurement in Model 2 (p = 0.175); however, the assumptions of the second AMH measurement and the global model were violated (p < 0.05).

**Figure 2:**
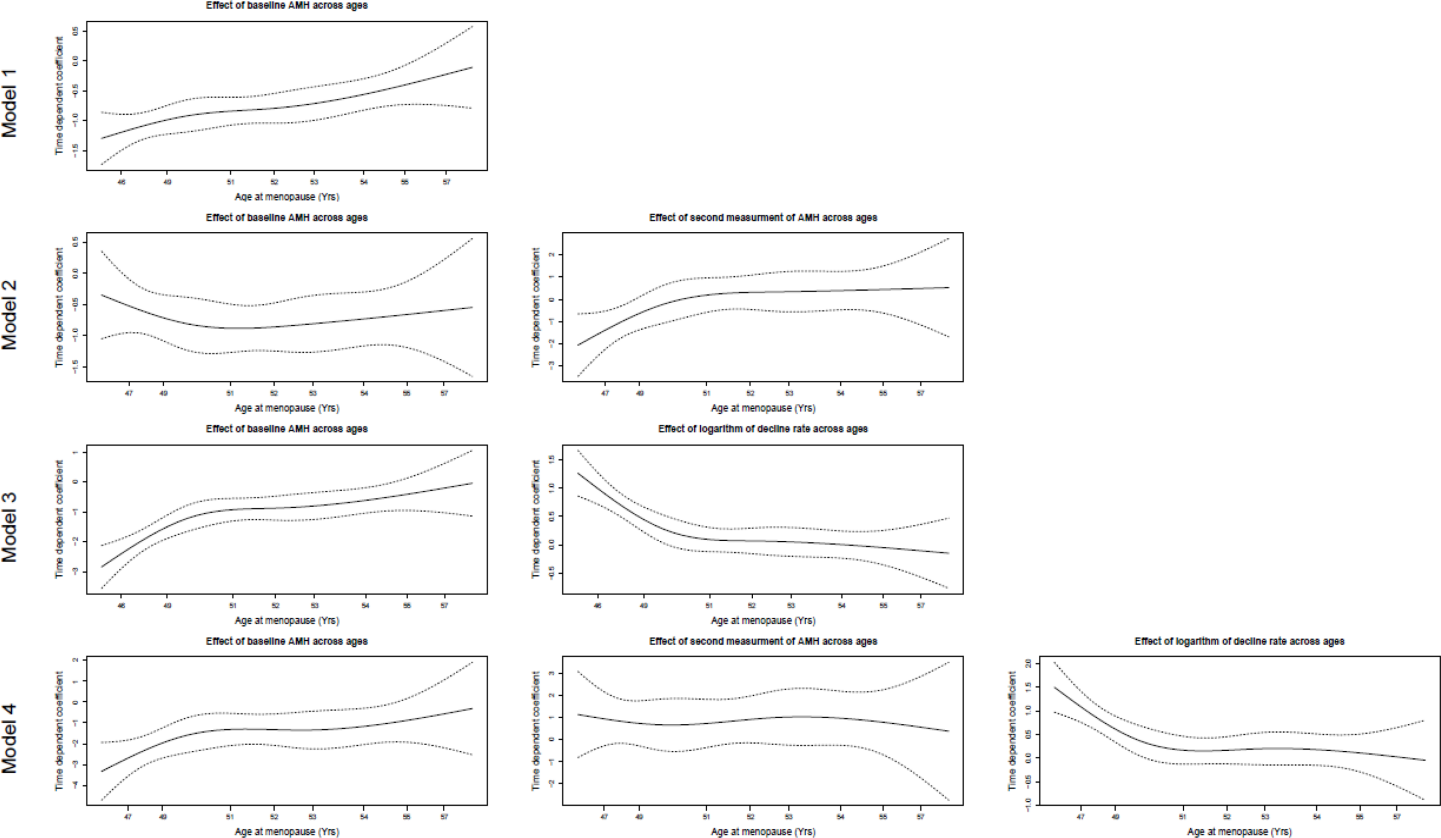
The visual assessment of proportional hazard assumption for Cox regression models. Model 1: The effect of baseline AMH measurement on survival time adjusted by age at baseline and storage time for baseline AMH. Model 2: The effects of baseline and second AMH measurements on survival time adjusted by age at baseline, storage times of baseline, and second AMH measure. Model 3: The effects of baseline AMH measurement and logarithm of AMH annual decline rate on survival time adjusted by age at baseline, storage times of baseline, and second AMH measure. Model 4: The effects of baseline AMH measurement, second AMH measurement, and logarithm of AMH annual decline rate on survival time adjusted by age at baseline, storage times of baseline, and second AMH measure.

**Figure 2** is a visual representation of the PH assumption in our models. Although the results showed that the PH assumption held true for the baseline AMH values in Models 2, 3, and 4, the related lines suggest deviations from this assumption. However, this test only considered linear time trends, while a higher order of relations may exist. A polynomial of degree 3 was suitable for Model 3 and Model 4. The VIFs did not exceed the cutoff value of 4 in the first three models; in other words, there was no significant collinearity requiring further exploration. However, the VIF for Model 4 was about 10 (9.89), which revealed a severe multicollinearity requiring model correction.

The results of the subgroup analysis for the prediction of early menopause are presented in **Table 2**. It was found that the AMH annual decline rate was the most effective predictor. Each unit increase in the logarithm of decline rate multiplied the risk of menopause by 2.38. Equivalently, the risk of early menopause increased by 10.8 with each unit increase in the AMH annual decline rate. Based on the C-statistic, the best model for predicting early menopause included the baseline AMH measurement and the AMH annual decline rate (C-statistic = 0.75); the hazard ratios for the baseline AMH and the annual AMH decline rate were 0.22 and 2.33 in this model, respectively. However, only Model 2, which included both the baseline and second AMH measurements, satisfied the PH assumption (p = 0.75). The VIF did not exceed the cutoff value of four in the first three models, while it was 12.91 in Model 4; therefore, inclusion of both AMH measurements and the logarithm of the decline rate in Model 4 caused considerable multicollinearity.

**Table 3** summarizes the results of our second approach, based on the time-dependent Cox regression analysis. Model 1 considered the AMH measurement at each time point as the primary predictor and was adjusted for the baseline age. According to this model, the risk of menopause decreased by 87% with each unit increase in the AMH value (ng/mL). Besides, the addition of AMH storage time to the model increased the C-statistic by 21%. Model 3 incorporated the logarithm of the annual AMH decline rate instead of rather than the AMH values; although the C-statistic was 84%, the hazard ratio was not significant.

**Table 3:** Summary statistics for the time-dependent Cox regression approach for prediction of menopause/early menopause according to single/repeated AMH measurements.

Variable	Menopause prediction during follow-up		Prediction of early menopause^€^	
	HR (95% CI)	C-statistic (95% CI)	HR (95% CI)	C-statistic (95% CI)
Model 1^*^				0.71 (0.61–0.96)
AMH value	**0.13 (0.04–0.48)**	0.64 (0.61–0.67)	0.07 (0.00–6.45)	
Model 2^**^				0.74 (0.64–0.95)
AMH value	**0.03 (0.003–0.22)**	0.85 (0.83–0.87)	0.09 (0.001–6.51)	
Model 3^**^				0.83 (0.75–0.91)
Log (annual AMH decline rate)	0.92 (0.84–1.01)	0.84 (0.83–0.85)	**2.35 (1.02–5.40)**	
Model 4^**^				0.72 (0.59–0.96)
AMH value	**0.03 (0.003–0.23)**		0.02 (0.00–11.60)	
Log (annual AMH decline rate)	1.00 (0.91–1.10)	0.85 (0.83–0.87)	2.77 (0.96–8.02)	

^*^Model 1: adjusted by age at baseline.

^**^Models 2, 3, and 4: adjusted by age at baseline and storage time of AMH.

Significant results are reported in bold. Annual AMH decline rate was calculated according to last AMH-baseline AMH)/time intervals (years), reported as AMH (ng/ml/year).

AMH, anti-Mullerian hormone; HR, hazard ratio.

^€^Early menopause was defined as menopause that happened at age < 45 years.

Finally, Model 4 included both AMH measurements and AMH annual decline rate; the hazard ratio for AMH was the same as Model 2, and the decline rate was still insignificant. Therefore, in the presence of AMH levels at each observation time, the decline rate did not add any information. Also, the subgroup analysis for the prediction of early menopause revealed similar results (**Table 3**). Moreover, in a comparison of two subjects with the same conditions and a 1-ng/ml difference in the AMH level, Model 2 indicated a 90% lower risk of early menopause. Under different conditions, Model 3 provided information about time variations in the AMH levels. Therefore, the risk of early menopause in women with a one-unit decline rate was 3.52 times higher than that of women with a stable AMH over a year.

## Discussion

The present study revealed that regardless of using various statistical approaches, repeated AMH measurements could improve the prediction of age at menopause or early menopause. It should be emphasized that models including both AMH level in each observation time and the annual decline rate of AMH cause multicollinearity issues, as they obscure the beneficial effects of multiple AMH measurements on improving the prediction of menopause. In comparison of Cox proportional-hazards and time-dependent regression models, the former was found to be less suitable, since the PH assumption was not satisfied for repeated measurements of AMH, while the time-dependent Cox regression model did not meet this assumption, and multicollinearity may not influence the results.

Previous studies have shown that a single AMH measurement cannot be a sensitive indicator for the prediction of age at menopause at an individual level (24–26). The majority of models assumed a steady rate of follicular atresia over time, resulting in a comparable decreasing pattern for each woman (17, 27). Although the level of AMH is primarily determined by the initial primordial follicle pool size (27), its level may be influenced by biological characteristics, reproductive factors, or environmental/lifestyle factors (9–14, 28), leading to a complex pattern of AMH decline.

Freeman et al., in the 14-year population-based Penn Ovarian Aging Stud, found that adding the change rate of AMH to the single AMH measurement or age alone could improve the estimation of time to menopause (17). Besides, de Kat et al., in the 20-year population-based Doetinchem Prospective Study, reported that the decline in age-dependent AMH levels did not follow a fixed pattern for each woman, as it varied significantly for individuals over time (15). It was hypothesized that adding the AMH change rate to a prediction model with a single AMH measurement might improve the prediction of age at menopause. However, studies using repeated AMH measurements have reported conflicting results, mainly due to the use of different statistical approaches and not meeting the essential preliminary assumptions.

Moreover, in a smaller subset of the TLGS population, including 266 women with a shorter average follow-up of 6.5 years, we found that multiple AMH measurements could provide an individualized prediction of age at menopause (18). However, with a larger sample size, a higher menopausal event rate, a longer follow-up of TLGS study (19), and also use of an accelerated failure time model with the Weibull distribution, we found that prediction of age at menopause could be improved if the annual decline rate was added to the model including AMH alone; the C-statistic increased from 70% to 78%, and the difference between the actual and predicted age at menopause decreased from -0.48 to -0.21 years (19).

In contrast, in another recent study, de Kat et al. reanalyzed the data of 2,434 premenopausal women from the population-based Doetinchem Cohort Study. Based on the time-varying Cox model, the AMH decline rate alone or in combination with age-specific AMH had a small added value; they concluded that knowledge of the AMH decline rate does not improve the prediction of menopause (16). Although this discrepancy may be partially explained by the heterogeneity of population characteristics and use of different statistical approaches, in the present study, we aimed to reexamine our dataset using time-varying Cox modeling to explore the factors that may partly explain these controversial findings.

The Cox proportional-hazards regression modeling is commonly used to analyze the survival data. However, the critical assumption of this model has been rarely examined in applied studies (4, 7, 29). According to the PH assumption, the hazard ratio should be constant over time; in other words, the hazard for one individual is proportional to the hazard for any other individual. Besides, the proportionality constant is independent of time (30). As the estimation technique in Cox regression models depends on the PH assumption, a violation may lead to an incorrect inference or underestimation of the hazard ratio (in case of an increase in the hazard proportion) (31, 32).

In the present study, we explored the adequacy of Cox models, using time-weighted score tests of the proportional-hazards hypothesis (33). The results revealed that the PH assumption was violated if we considered only one value for all AMH measurements, as reported by de Kat et al. (16). In other words, the hazard ratio varies with time and depends on the magnitude of change. Therefore, parametric models, extended Cox regression for time-varying variables, and joint modeling have been suggested (19, 29, 31, 34). The time-dependent Cox regression model has been extended for when a variable cannot satisfy the PH assumption. This model is more appropriate for covariates that vary as a function of time (external covariates) (35). Time dependence is considered in the model by entering different values of covariates and clustering them for the individual. Therefore, the model easily handles variations in covariates across time (36).

On the other hand, the multicollinearity of dependent variables in all regressions can cause serious problems, such as undermining the statistical significance of variables (37). In the study by de Kat et al., while a time-dependent Cox regression model was used with an appropriate C-statistic (0.70), adding the AMH annual decline rate did not improve the C-statistic, and the hazard ratio of this rate changed the effects (from 1.36 in the crude model to 0.98 in the adjusted model by AMH levels) (16). This discrepancy may be partly explained by the inclusion of both AMH values and their variations in the time-dependent Cox model, because it led to multicollinearity which did not improve the predictions.

The main strength of the present study was its methodology, as it was a large population-based research with long-term follow-ups using advanced statistical methods while meeting the essential assumptions. Menopause occurred in more than half of the participants during the follow-ups, which improved the reliability of our predictions. Moreover, the intra- and inter-assay variability in AMH measurements was minimal due to the use of a single AMH kit and performing all measurements in a single laboratory.

This study also had some limitations. First, other ovarian aging markers, such as the antral follicle count, were not measured. Second, we used the Gen II assay (Beckman Coulter, CA, USA) for the AMH measurements. Compared to the picoAMH assay (Ansh Labs LLC, TX, USA), the Gen II assay has less sensitivity, particularly in the lower range of AMH, resulting in a higher limit of detection (38); however, there seems to be a significant correlation between these two assays (39). Third, use of stored frozen samples for AMH measurements may potentially affect the stability of AMH. However, it has been documented that storage at -80°C and episodes of thawing have little effects on the AMH level, according to the AMH Gen II assay (40, 41). Fourth, our results remained unchanged after adjustment for the sample storage time as the confounding variable. However, it remains unclear whether the current findings obtained from women with a previously normal fertility can be translated to infertile women in whom the ovarian aging process may be compromised. Overall, 9.4% of our population were younger than 40 years, and one-third of them did not reach menopause; however, the occurrence of menopause was adequate for achieving reliable results in this study.

In conclusion, this study confirmed that multiple AMH measurements can improve the individualized prediction of physiological menopause age compared to single AMH measurements. Also, different alternative statistical approaches can offer the same interpretations of findings if the essential assumptions are met. However, use of multiple AMH measurements for the prediction of age at menopause in clinical practice still requires further investigation.

## Data Availability Statement

The raw data supporting the conclusions of this article will be made available by authors, upon request, without undue reservation.

## Ethics Statement

The studies involving human participants were reviewed and approved by the Ethics Committee of Research Institute for Endocrine Sciences, Shahid Beheshti University of Medical Sciences, Tehran, Iran. The patients/participants provided their written informed consent to participate in this study.

## Author Contributions

Conceptualization, FRT, SB-G, FA. Methodology, FRT, AS. Software, AS. Validation, FRT, FA. Formal analysis, AS, FRT. Investigation, SB-G, FF, MT. Data curation, FRT, FF, SB-G. Writing—original draft preparation, FRT, SB-G, AS. Writing—review and editing, FRT, FF, FA, MT. Supervision, SB-G, FRT. Project administration, FR. All authors contributed to the article and approved the submitted version.

## Funding

This research projected was funded by the Shahid Beheshti University of Medical Sciences (Grant no. 5).

## Conflict of Interest

The authors declare that the research was conducted in the absence of any commercial or financial relationships that could be construed as a potential conflict of interest.

## Publisher’s Note

All claims expressed in this article are solely those of the authors and do not necessarily represent those of their affiliated organizations, or those of the publisher, the editors and the reviewers. Any product that may be evaluated in this article, or claim that may be made by its manufacturer, is not guaranteed or endorsed by the publisher.
